# Functional identification of two novel variants and a hypomorphic variant in *ASS1* from patients with Citrullinemia type I

**DOI:** 10.3389/fgene.2023.1172947

**Published:** 2023-07-07

**Authors:** Jing Liu, Zhongjie Wang, Huiming Yan, Yanling Teng, Qingxin Shi, Jing Chen, Wanglan Tang, Wenxian Yu, Ying Peng, Hui Xi, Na Ma, Desheng Liang, Zhuo Li, Lingqian Wu

**Affiliations:** ^1^ Department of Medical Genetics, Maternal and Child Health Hospital of Hunan Province, Changsha, Hunan, China; ^2^ National Health Commission Key Laboratory of Birth Defects Research, Prevention and Treatment, Changsha, Hunan, China; ^3^ Center for Medical Genetics, Hunan Key Laboratory of Medical Genetics, Hunan Key Laboratory of Animal Models for Human Diseases, School of Life Sciences, Central South University, Changsha, China; ^4^ Laboratory of Molecular Genetics, Hunan Jiahui Genetics Hospital, Changsha, Hunan, China

**Keywords:** *ASS1*, Citrullinemia type I, urea cycle disorder, hypomorphic variant, enzyme activity

## Abstract

**Background:** Citrullinemia type I (CTLN1) is a rare autosomal recessive inborn error of the urea cycle caused by mutations in the gene encoding the arginosuccinate synthetase (ASS1) enzyme. Classic CTLN1 often manifests with acute hyperammonemia and neurological symptoms. Molecular genetic testing is critical for patient diagnosis.

**Methods:** Three unrelated families with clinically suspected CTLN1 were included in this study. Potential pathogenic variants were identified using whole exome sequencing (WES) and validated using Sanger sequencing. Western blotting, quantitative PCR, immunofluorescent staining, and ELISA were used to assess functional changes in candidate *ASS1* variants.

**Results:** Five variants were identified, two of which were novel, and one has been reported, but its pathogenicity was not validated. The novel variant c.649-651del (p.P217del) and the 5′UTR variant (c.-4C>T) resulted in a decrease in *ASS1* expression at both the protein and transcription levels. The other novel variant, c.1048C>T (p.Q350*), showed a marked decrease in expression at the protein level, with the formation of truncated proteins but an increased transcription. Both c.649_651del (p.P217del) and c.1048C>T (p.Q350*) showed a highly significant reduction in enzyme activity, while c.-4C>T had no effect.

**Conclusion:** We identified two novel variants and a hypomorphic non-coding variant in *ASS1* and validated the pathogenicity using functional studies. Our findings contribute to expanding the spectrum of *ASS1* variants and understanding the genotype-phenotype relationships of CTLN1.

## 1 Introduction

Citrullinemia type I (CTLN1, MIM#215700) is a rare autosomal recessive disorder of the urea cycle caused by a defect in the function of argininosuccinate synthase resulting from mutations in the *ASS1* gene (BEAUDET et al., 1986). The prevalence of CTLN1 is estimated to be one in 100,000 (RATNER, 1973; BEAUDET et al., 1986; Ah Mew et al., 1993). Life threatening hyperammonemia, markedly elevated plasma citrulline levels, low plasma arginine levels, poor feeding, neurological impairments and orotic aciduria are characteristics of the classic CTLN1 (Quinonez et al., 1993; BEAN et al., 2016). The diagnosis of CTLN1 can be confirmed by decreased ASS1 enzyme activity measured in cultured fibroblasts or by identifying biallelic pathogenic variants of the *ASS1* gene (Quinonez et al., 1993; BURKI, 2016).

The human *ASS1* gene is located on the long arm of chromosome 9 within the band 9q34.11–9q34.12 and contains 16 exons encoding 412 amino acids (HABERLE et al., 2002). The *ASS1* gene has 10–14 homologous copies scattered across the human genome; however, the sequence on chromosome 9 seems to be the only basis for a functional protein in which mutations associated with CTLN1 have been found (ENGEL et al., 2009). ASS1 functions as a rate-limiting enzyme in the third step of the urea cycle to catalyze the formation of argininosuccinate (from citrulline and aspartic acid), which is mainly expressed in periportal hepatocytes of the liver (RATNER, 1973). The enzyme contains three domains: a nucleotide-binding domain containing an “N-type” (ATP binding domain, from the N-terminal to Pro^165^), a synthetase domain (Val^166^ to Arg^359^), and a C-terminal oligomerization domain (Gln^360^ to C-terminal) (GOTO et al., 2002; KARLBERG et al., 2008; ENGEL et al., 2009).

The clinical phenotype is highly heterogeneous, based on the time of onset, CTLN1 can be classified into classic neonatal onset and late onset. The phenotype of the classical neonatal onset CTLN1 is described above. In the milder late-onset CTLN1, patients may show recurrent neurological symptoms such as lethargy and mental retardation, or psychiatric symptoms, or patients may remain asymptomatic with only a biochemical phenotype that may be detected during newborn screening (DIEZ-FERNANDEZ et al., 2017). Thus molecular genetic testing is useful for diagnosing CTLN1. To date, 164 distinct mutations have been identified in the *ASS1* gene in the Human Gene Mutation Database (HGMD), most of which are missense mutations. The mutations are distributed throughout exons 3–15, but most are identified in exons 5, 12, 13, and 14 (DIEZ-FERNANDEZ et al., 2017). To date, only 11 Chinese patients have been documented as positive for CTLNI based on molecular genetic studies (HU et al., 2011; WEN et al., 2014; WU et al., 2014; XIE et al., 2014; SUN et al., 2018; Lin et al., 2019).

In this study, we identified three neonates with CTLN1 using molecular analysis of the *ASS1* gene and biochemical characteristics. The clinical features of the patients and follow-up information are summarized. Furthermore, two novel variants of *ASS1* [c.649_651del (p.P217del) and c.1048C>T (p.Q350*)] and a 5′UTR variant (c.-4C>T) were validated for pathogenicity, thereby expanding the mutation spectra of the *ASS1* gene.

## 2 Materials and methods

### 2.1 Subjects and ethics statement

From 2018 to 2022, three unrelated Chinese families were recruited to participate in the study at the Maternal and Child Health Hospital of Hunan Province and Hunan Jiahui Genetics Hospital. Detailed clinical data and family histories were obtained from hospital-issued medical records and follow-ups, and the three probands had varying degrees of CTLN1. Consent was obtained from the guardians of the patients, and approval was obtained from the Ethics Committee of the Center for Medical Genetics, Central South University, Hunan, China.

### 2.2 Molecular genetic analysis

Genomic DNA was extracted from whole blood samples of the probands and their parents using the Qiagen Blood DNA mini kit (Qiagen, Hilden, Germany) or Quickgene DNA Whole Blood Kit L (FUJIFILM, Tokyo, Japan) according to the manufacturers’ instructions (LI et al., 2023). Whole exome sequencing (WES) was performed on the available DNA from all members of the three families (Genuine Diagnostics and Berry Genomics Inc., China). Exons were captured using Nano WES 2.1 (Berry Genomics Inc., Beijing, China) and sequenced using Novaseq6000 platform (Illumina, San Diego, United States) with 150 bp paired-end reads. The sequencing reads were aligned to the human reference genome (hg19/GRCh37) using Burrows–Wheeler Aligner tool and PCR duplicates were removed by using Picard v1.57 (http://picard.sourceforge.net/). Verita Trekker^®^ Variants Detection System by Berry Genomics and the third-party software GATK (https://software.broadinstitute.org/gatk/) were employed for variant calling. Variant annotation and interpretation were conducted by ANNOVAR and the Enliven^®^ Variants Annotation Interpretation System authorized by Berry Genomics (Wang et al., 2010).Candidate variants were confirmed using Sanger sequencing by ABI 3730XL (Thermo Fisher Scientific, MA, United States), the protocol used for DNA amplification is shown in the Supplementary Protocol S1. The primers to confirm mutations were those used in a previous report (KARLBERG et al., 2008). Pathogenicity analysis of the variants was performed per the American College of Medical Genetics and Genomics (ACMG) guidelines (Richards et al., 2015).

### 2.3 *In silico* analysis

To explore the potential functional impact of different mutations by using multiple bioinformatic tools, including SIFT (http://blocks.fhcrc.org/sift/SIFT.html), Varcards (http://varcards.biols.ac.cn/), MutationTaster (http://www.mutationtaster.org/), and CADD (http://cadd.gs.washington.edu/score). The degree of conservation was analyzed using the MEGA11 software (Tamura et al., 2021), and amino acid sequences were obtained from NCBI. Based on the DPB database, the crystal structures of the ASS1 wild-type and mutant proteins were built using SWISS-MODEL (DVORYAKOVA et al., 2022). To investigate changes in hydrogen bonding between amino acids as well as the surface charge of proteins, 3D structural models of wild-type and mutant proteins, constructed using the Swiss model, were visualized using PyMOL software (DeLano, 2002).

### 2.4 Plasmid construction and transfection

We performed functional studies on the novel small deletion c.649_651del (p.P217del), the nonsense mutation c.1048C>T (p.Q350*), and the previously reported mutation c.-4C>T. A wild-type cDNA plasmid was purchased from YOU Bio-sciences (YOU Biosciences Inc., Hunan, China) and was inserted into a pcDNA3.1-3 × Flag-C carrier vector (ZHOU et al., 2022). Site-directed mutagenesis was performed using Mut Express II Fast Mutagenesis Kit V2 (Vazyme). Primers used for site-directed mutagenesis are listed in Supplementary Table S1. All wild-type and mutant plasmids were verified using Sanger sequencing, and no other mutations were found in the 293T cell line used for the *in vitro* analyses. 293T cells were cultured in six-well plates for transfection experiments. When the cell density reached 70%–80%, the wild-type or mutant *ASS1* plasmids were transfected into 293T cells using Lipofectamine™ 3000 Transfection Reagent (Thermo Fisher Scientific, Shanghai, China) containing 2 μg of the plasmid to achieve an optimal transfection effect. In addition, the empty vector was transfected into cells as a blank control. All transfections were harvested after 48 h.

### 2.5 Real-time quantitative PCR

Total RNA was extracted from the transfected 293T cells using the TRIzol reagent (Invitrogen, Carlsbad, CA, United States) and the concentration of RNA was measured by NanoDrop 1000 (Thermo Fisher Scientific, MA, United States). Briefly, 1000 ng of RNA was reverse transcribed to cDNA using Thermo Scientific RevertAid RT (Thermo Fisher, Carlsbad, CA, United States). qPCR was performed using Maxima SYBR Green qPCR Master Mix (Thermo Fisher) and an ASA-9600 Real-Time PCR System (Suzhou Baiyuan Gene Technology Co., Ltd., Suzhou, China) (ZHOU et al., 2022). The primers used were as follows: *ASS*1-qPCR-F: 5′-TTC​CCT​CAG​GCT​GAA​GGA​ATA​T-3′ and *ASS1*-qPCR-R: 5′-TCA​TCC​TTG​TAG​TCG​ATG​TCA​T-3′. We used the housekeeping gene *β-Actin, TBP*, and *HPRT* as internal control genes. Relative gene expression levels were determined using the 2^−ΔΔCT^ method and normalized to the mRNA levels of the housekeeping gene *β-Actin*, the processing of qPCR data are listed in the Supplementary Table S2.

### 2.6 Western blot

Total protein was extracted according to the standard operating procedure using RIPA (Beyotime, Jiangsu, China) and Trixon-X100 (Beyotime). Polyacrylamide gel electrophoresis experiments were then performed on the extracted proteins. The primary anti-ASS1 antibody (Abcam, Cambridge, United Kingdom) was diluted to 1:1000, and the anti-lamin b1 antibody (Proteintech, Chicago, United States) used as an internal reference was diluted to 1:2000.

### 2.7 Immunofluorescence analysis

293T cells were seeded in 24-well plates and cultured in an incubator at 37°C and 5% CO_2_. When the cell density reached 30%–40%, wild-type or mutant *ASS1* plasmids were transfected into 293T cells using Lipofectamine™ 3000 Transfection Reagent (Thermo Fisher Scientific, Shanghai, China). In line with the standard experimental procedure, the cells were harvested after 48 h, fixed with 4% paraformaldehyde at room temperature for 15–20 min, permeabilized with 0.5% TritonX-100 (PBS formulation) for 20 min, and then blocked with 5% bovine serum albumin (BSA) for 30 min. They were then incubated with primary antibody (1:300) in 5% BSA overnight at 4°C. The next day, the cells were incubated with a secondary antibody (Dylight 488 Mouse Anti-Goat antibody) for 1 h and then with DIPA for 10 min, and then shielded using Fluoromount™ Aqueous Mounting Medium (Sigma Aldrich, St. Louis, MO, United States). Finally, TCS SP5 laser confocal microscopy (Leica, Wetzlar, Germany) was used to acquire the immunofluorescence images.

### 2.8 Enzyme-linked immunosorbent assay (ELISA)

The activity of the ASS1 enzyme was determined using the Human ASS1 ELISA kit (Meimian, Jiangshu, China), which is based on the double-antibody sandwich method. Cells were collected 48 h after transfection, and total protein was extracted. The standards and samples were diluted, and each sample was added to triplicate antibody-coated wells while blank wells were also set. The wells were incubated at 37°C for 30 min before washing five times with a concentrated washing solution. The HRP-conjugate reagent was then added, and the wells were again incubated for 30 min. The wells were washed five times (10 min each time), followed by the addition of chromogen solutions A and B in the dark, after which the stop solution was added to terminate the reaction. The absorbance of each well was measured sequentially at 450 nm. Finally, the enzyme activity in the sample was calculated using a linear regression equation. The correlation coefficient R^2^ of sample linear regression and expected concentration was above 0.95, and the coefficient of variation within and between batches should be less than 10% and 12%, respectively (ZHOU et al., 2021).

### 2.9 Statistical analysis

All data analyses were performed using Prism 6 software (GraphPad, La Jolla, CA, United States), and the significance of the differences between groups was calculated using the *t*-test, where differences were considered statistically significant at *p* < 0.05.

## 3 Results

### 3.1 Clinical features of patients and follow-up information

Patient 1 was a female neonate born at a gestational age of 40 weeks and 2 days via cesarean section. Her birth weight was 3100 g. She was the first child of consanguineous parents of Chinese descent. There was no significant family history of inherited metabolic diseases. The newborn screening (NBS) test revealed elevated citrulline levels [690.57 μmol/L; reference range (RR), 7.0–30 μmol/L] and decreased arginine levels (0.6 μmol/L; RR, 1.3–42 μmol/L) (Table 1). At 30 days after birth, plasma amino acid analysis showed an increased level of citrulline (1780.87 μmol/L; RR, 7.0–30 μmol/L), normal arginine levels (11.11 μmol/L; RR, 1.3–42 μmol/L), and ammonia at 57 μmol/L (RR, 8.0–47 μmol/L) (Table 1). Urinary organic acid analysis via gas chromatography-mass spectrometry (GC-MS) revealed elevated levels of *a*-ketoglutaric acid, glycerin, 3-hydroxyproponic acid, alanine, pyruvate, and 4-hydroxyphenylacetic acid, elevated Cit/Arg and Cit/Phe ratios, but elevated levels of orotic acid were not observed. The patient occasionally vomits, occasionally shakes his head during sleep, and occasionally experiences lower limb tonicity when awake. Genetic analysis was performed based on the biochemical results, which indicated the diagnosis of CTLN1. Since then, she has been managed with dietary protein restriction, sodium phenylbutyrate, and arginine supplementation. Her plasma citrulline levels remained high (1650 μmol/L at 17 months and 1090 μmol/L at 20 months), and her ammonia levels were slightly elevated (84 μmol/L at 17 months and 100 μmol/L at 20 months) (Table 1). We investigated her GESELL score at one and a half years, which was as follows: developmental quotient (DQ), 83; fine motor, 71; gross motor, 71; adaptability, 78; language, 102; and social skill, 92. She is currently three and a half years old and still shows biochemical abnormalities (citrulline, 935.88 μmol/L; ammonia, 50 μmol/L) (Table 1) but exhibits normal growth and development. Her GESELL score at three and a half years was as follows: DQ, 78; fine motor, 100; gross motor, 68; adaptability, 71; language, 82; and social skill, 71.

**TABLE 1:** Clinical features and follow-up information of patients with Citrullinemia type 1.

Patient ID	Age at onset	Sex	Follow-up clinical features												Genotypes	Out come
			Citrulline (µmol/L)			Ammonia (µmol/L)			Cit/Arg			Cit/Phe				
			References: 7–30			References: 18–72			References: 0.40–10			References: 0.13–0.75				
1	1 m	Female	1780.87↑ (1 m)	1090.9↑ (20 m)	935.88↑ (42 m)	57 (1 m)	100↑ (20 m)	50 (42 m)	160.22↑ (1 m)	50.55↑ (20 m)	246.28↑ (42 m)	40.92 (1 m)	30.6↑ (20 m)	25.92↑ (42 m)	c.649_651del	Alive
															c.1048C>T	
2	1 m	Male	62.31↑ (1 m)	57.31↑ (14 m)	56↑ (17 m)	20 (1 m)	38 (14 m)	19 (17 m)	2.03 (1 m)	2.08 (14 m)	2.1 (17 m)	2.42↑ (1 m)	1.75↑ (14 m)	1.8↑ (23 m)	c.-4C>T	Alive
															c.910C>T	
3	2 day	Male	N/A	N/A	N/A	197↑ (2 days)	N/A	N/A	N/A	N/A	N/A	N/A	N/A	N/A	c.847G>A	Died
															c.1048C>T	

Note. m, month; d, day; (1 m), Clinical features at 1 month old; “↑“, Represents exceeding the reference value; NA, Not obtainable,”—“, No family history.

Patient 2 was a male, born at a gestational age of 38 weeks and 3 days, with a birthweight of 3,300 g after an uneventful pregnancy. He was the second child of consanguineous Chinese parents. He had a healthy older sister aged 6 years. There was no notable family history of inherited metabolic diseases. The initial NBS results showed a slightly elevated citrulline level with increased ratios of citrulline/arginine and citrulline/phenylalanine. Plasma amino acid analysis showed slightly increased levels of citrulline varying from 36.12 μmol/L to 101.42 μmol/L (RR, 7.0–30 μmol/L), but ammonia levels were normal (Table 1). At 30 days after birth, a repeated plasma amino acid analysis showed an increased citrulline level (62.31 μmol/L; RR, 7.0–30 μmol/L) (Table 1), but GC-MS analysis presented no abnormalities. The patient was subjected to normal feeding and exhibited normal developmental milestones until the age of 2 years.

Patient 3 was a male born via spontaneous vaginal delivery. He was quickly transferred to the neonatal intensive care unit 24 h after birth due to a poor response. Routine laboratory tests showed hyperammonemia with ammonia levels of 197 mmol/L (normal range: 18–72 mmol/L) (Table 1), and MSMS analysis was not performed. He was treated with fasting, blood purification, and arginine supplementation to reduce blood ammonia levels. A repeat examination revealed that the blood ammonia level was elevated again (no data), and the patient died 3 days after birth. He was the second child of unrelated Chinese parents. His mother had previously had a spontaneous abortion in the first trimester, and no genetic testing was performed.

### 3.2 Variants detected by WES and sanger sequencing

Patient 1 was compound heterozygous for two variants, c.649_651del/p. P217del (Chr9:133352309-133352311) and c.1048C>T/p.Q350* (Chr9:133370331), in *ASS1* (NM_000050.4); both parents were heterozygous for these variants. They were also absent from the dbSNP, 1000 Genomes, HGMD, ClinVar, and LOVD databases. According to the ACMG guidelines, c.649_651del (p.P217del) was classified as a variant of uncertain clinical significance, and c.1048C>T (p.Q350*) was classified as a likely pathogenic variant (Table 2).

**TABLE 2:** Variants identified in three patients and related information.

Patient ID	Exon	Parental origin	Genomic position	Nucleotide substitutions (NM_000050. 4)	Amino acid change (NP_000041.2)	Mutation type	Mutation taster	CADD	SIFT	Variant classification	References
1	10	P	chr9:133352309-133352311	c.649_651del	p.P217del	Small delection	Disease causing	N/A	N/A	VUS: PM2_Supporting + PM4	This study
	14	M	chr9:133370331	c.1048C>T	p.Q350*	Nonsense	Disease causing	Damaging	N/A	LP: PVS1+PM2_Supporting	This study
2	3	M	chr9:130452225	c.-4C>T (rs138350285)	—	5′UTR	N/A	N/A	N/A	VUS: PM2_Supporting + PM3	Engel K et al., 2009
	13	P	chr9:130489404	c.910C>T (rs121908642)	p.R304W	Missense	Disease causing	Damaging	Damaging	P: PM3_VeryStrong + PS3+PM2+PP3	Diez-Fernandez et al., 2017
3	13	P	chr9:130489341	c.847G>A (rs765338121)	p.E283K	Missense	Disease causing	Damaging	Damaging	LP: PM3_strong + PM2_supporting + PP1+PP3	Gao et al., 2003
	14	M	chr9:133370331	c.1048C>T	p.Q350*	Nonsense	Disease causing	Damaging	N/A	LP: PVS1+PM2_Supporting	This study

Note. P, paternal; M, Maternal.

Patient 2 was compound heterozygous for two variants, c.-4C>T and c.910C>T (p.R304W), in *ASS1* (NM_000050.4); both parents were heterozygous for these variants. The C.-4C>T variant is located in a non-coding region. Although the variant was identified in only two patients, no evidence of pathogenicity was observed. Thus, C.-4C>T is still classified as a variant of uncertain clinical significance. The c.910C>T variant has been identified in multiple patients in other countries, and this is the first report in China that classifies it as a pathogenic variant (Table 2).

Patient 3 was compound heterozygous for two variants, c.1048C>T (p.Q350*) and c.847G>A (p.E283K), in *ASS1* (NM_000050.4); both parents were heterozygous for these mutations. The c.1048C>T (p.Q350*) variant was the same as that in patient 1, and the c.847G>A (p.E283K) variant was identified in multiple patients previously and was classified as a likely pathogenic variant (Table 2).

**FIGURE 1:**
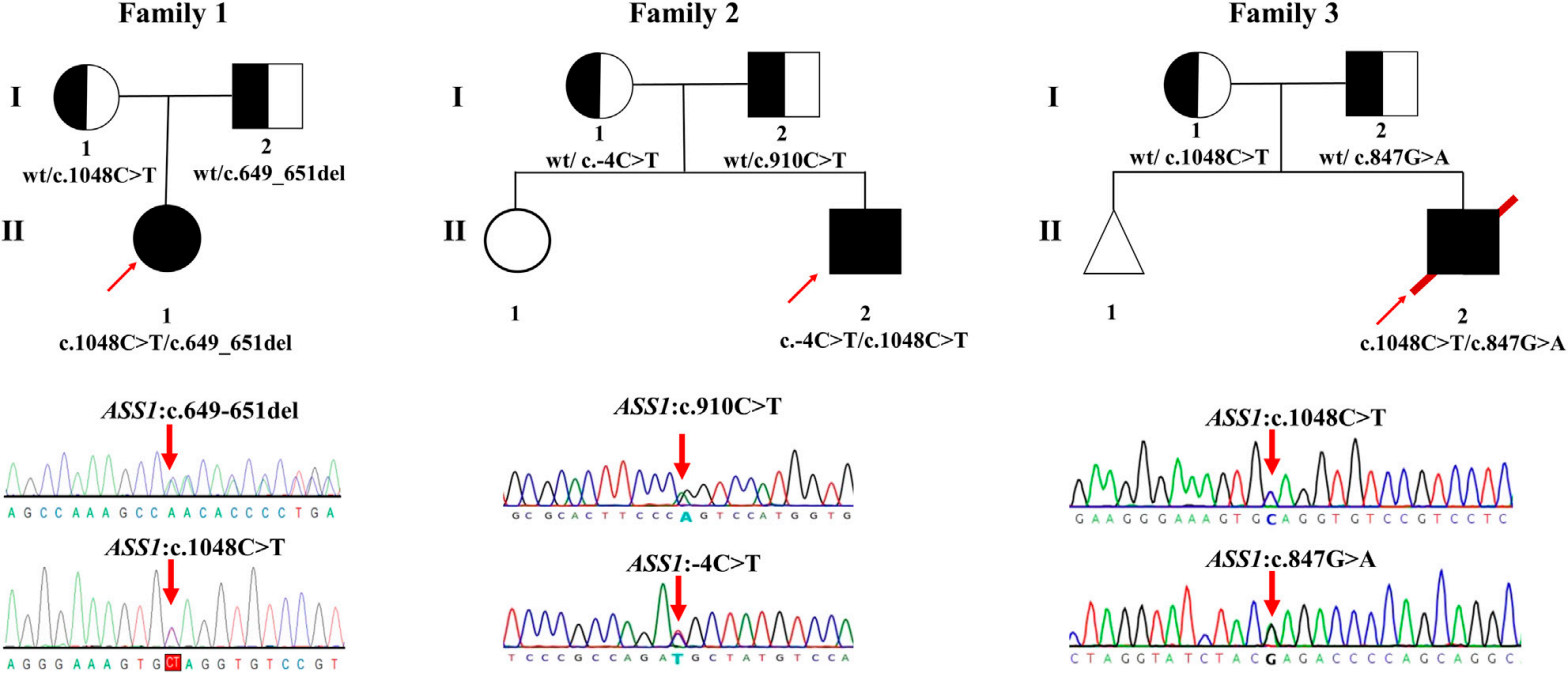
Pedigrees of the families investigated in this study. Sanger sequencing traces of probands 1–3 (corresponding to patient 1–3, all patients are compound heterozygote). The square and circle indicates the male and female respectively, the arrow represents the proband, the black background represents patients, the white background represents unaffected individuals, the half-black and the half-white background represents carriers, the triangle represents a fetal abortion, and the red slanted line means death.

Briefly, by analyzing WES data, five variants of *ASS1* were identified in three unrelated families and confirmed by Sanger sequencing (Figure 1), the details of which are displayed (Table 2). Because the three variants c.649_651del (p.P217del), c.1048C>T (p.Q350*), and C.-4C>T were novel or still classified as a variant of uncertain significance, further *in silico* analysis and functional experiments were conducted to confirm the functional changes and pathogenicity of the variants.

### 3.3 *In silico* analysis

**FIGURE 2:**
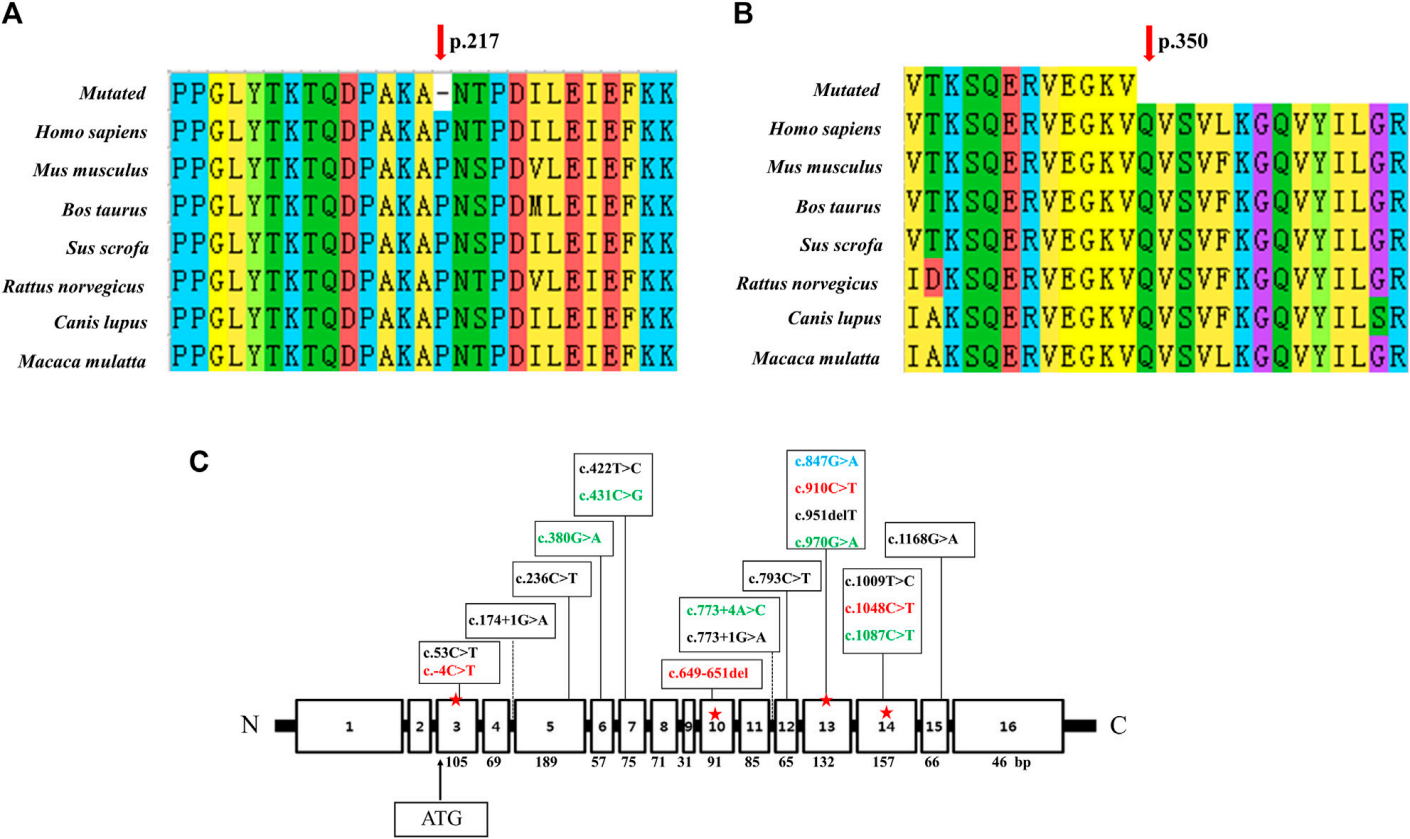
Conservation analysis of two novel variants and linear map of the *ASS1* gene with mutations reported in Chinese populations. **(A)** The variant c.649_651del p. (P217del) affects a highly conserved amino acid P217 in ASS1 orthologs. **(B)** The variant c.1048C>T p. (Q350*) affects a highly conserved amino acid Q217 in ASS1 orthologs. **(C)** Exon sizes are mentioned below each exon box. The arrow indicates the location of the start codon. The mutations marked in green were detected twice in 11 reported Chinese patients (HU et al., 2011; WEN et al., 2014; WU et al., 2014; XIE et al., 2014; SUN et al., 2018; Lin et al., 2019), and the mutations marked in red were found in our patient (c.649_651del was from patient 1, c.-4C>T and c.910C>T were from patient 2, c.1048C>T was detected both in patient 1 and 3). c.847G>A marked in blue was detected in both patient 3 and one of the 11 reported Chinese patients.

**FIGURE 3:**
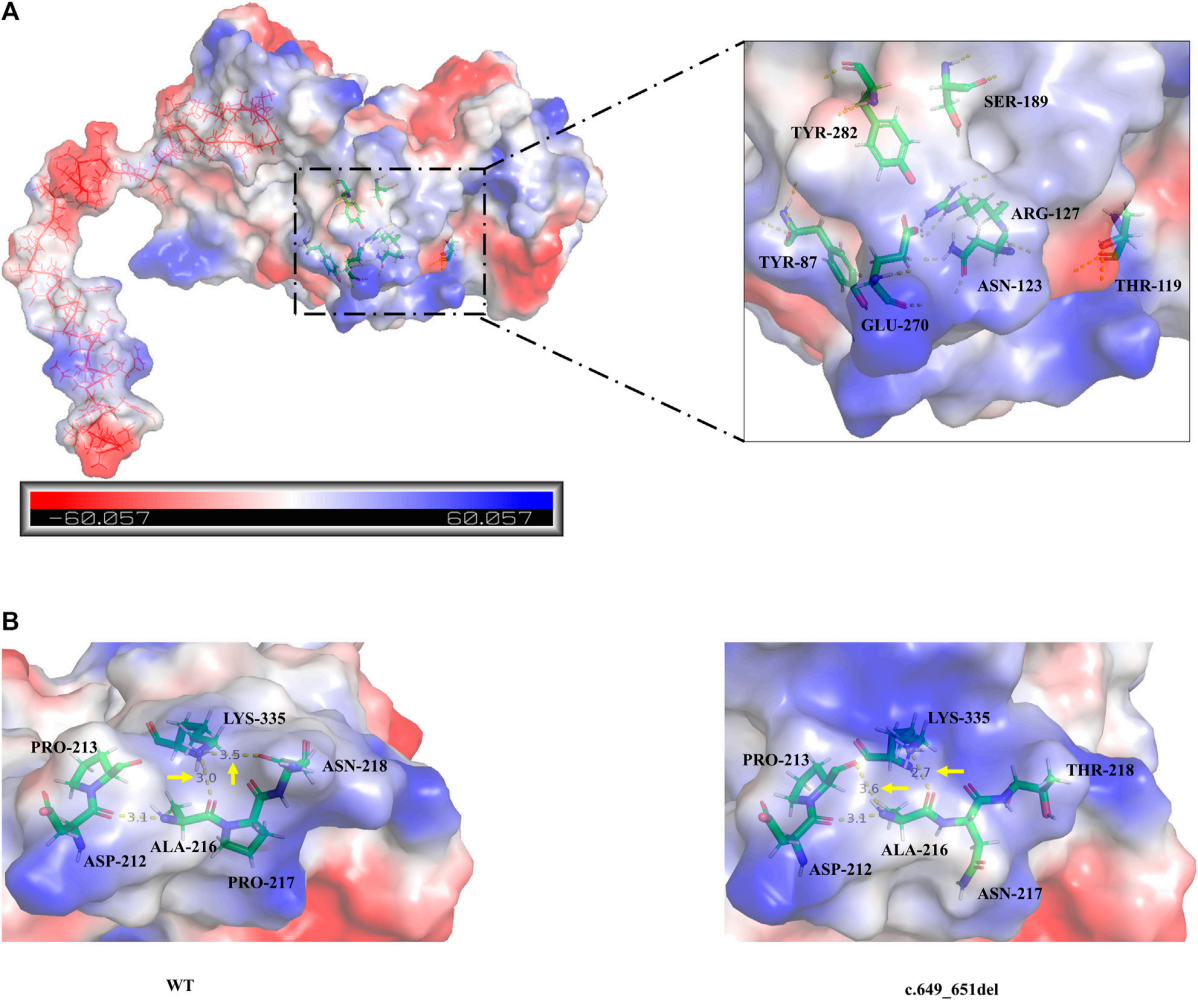
**(A)** The overall three-dimensional structural model of the wild-type protein. c.1048C>T variant leading to the loss of 63 amino acids is shown as the continuous red line. The enzyme active sites bound to the substrate (Tyr87, Thr119, Asn123, Arg127, Ser189, Glu270, and Tyr282) are represented in a colored rod structure, and the surface charge distribution is shown in the outer layer, with blue representing positive charge, red representing negative charge, and white representing neutral charge. **(B)** A partial model of the wild-type and c.649_651del variants. Amino acids are shown as colored rod structures, and the hydrogen bonds between amino acids are marked by yellow dashed lines.

Analysis of the degree of conservation showed that the locations of the variants c.649_651del (p.P217del) and c.1048C>T (p.Q350*) were highly conserved across a broad range of species (Figures 2A, B), and both were located in the synthetase structural domain. We developed a model of wild-type protein containing enzyme active sites (KARLBERG et al., 2008) (Figure 3A), *in silico* protein modeling revealed that the c.1048C>T variant causes a loss of 63 amino acids, including the entire C-terminal oligomerization domain (Gln360 to C-terminal) (WEN et al., 2014; XIE et al., 2014; SUN et al., 2018) (Figure 3A).In the c.649_651del mutant model, the length of the hydrogen bond between alanine at position 216 and lysine at position 335 was shortened, and a new hydrogen bond between alanine at position 216 and proline at position 213 was formed. In addition, the hydrogen bond between lysine at position 335 and threonine at position 218 was deleted (LI et al., 2023b)^.^ Meanwhile, a change in protein surface charge was predicted in the c.649_651del mutant protein model (Figure 3B).

### 3.4 Variants alter the *ASS1* transcription and protein levels

**FIGURE 4:**
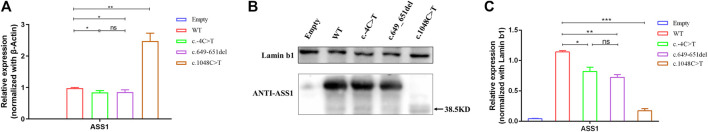
**(A)** qRT-PCR showing that the variants were altered compared to the wild type, with a decrease in the c.-4C>T and c.649_651del variants and a significant increase in the c.1048C>T variant. **(B)** WB confirming that c.1048C>T formed a truncated protein with a molecular weight of approximately 38.5 kDa **(C)** Quantification of WB showed that compared to the wild type, the variant had a decrease in protein expression levels compared to wild-type (*t*-test, *:*p* < 0.05; **:*p* < 0.01; ***:*p* < 0.001). All experimental results are representative of three independent tests.

To explore the functional effects of the three variants, 293T cells were transfected with the empty, WT, and mutant plasmids. The empty group was used as a blank control, and the data of standardization of *β-Actin* is provided (Supplementary Figure S1). The qRT-PCR results revealed that compared with the wild type, the c.-4C>T and c.649_651del variants led to a decrease in the transcription level, but the c.1048C>T variant resulted in a significant increase. Meanwhile, there was no difference in the expression of *TBP* and *HPRT* between the groups (Figure 4A). Western blot results showed that the c.-4C>T and c.649_651del variants resulted in similar protein levels, while the c.1048C>T variant caused a truncation of ASS1 (with a molecular weight of approximately 38.5 kDa) and drastically decreased the protein level (Figures 4B, C). To further clarify whether variants alter the subcellular localization of ASS1, immunofluorescence staining was performed, and the results demonstrated that all three mutant proteins remained evenly distributed in the cytoplasm without abnormal aggregation or significant alteration to the subcellular localization (Supplementary Figure S2).

### 3.5 Variants reduce ASS1 enzyme activity

**FIGURE 5:**
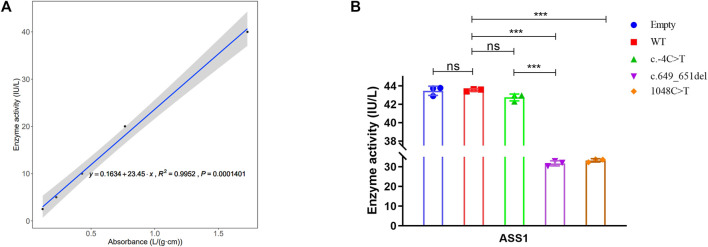
Detection of ASS1 enzyme activity by ELISA for the variants. **(A)** Linear regression curve between absorbance and enzyme activity (Five concentrations of standards). **(B)** Comparison of enzyme activity between WT and different variants (*t*-test, *:*p* < 0.05; **:*p* < 0.01; ***:*p* < 0.001).

To explore the effect of these variants on enzyme activity, we performed ELISA. The correlation coefficient R^2^ of the sample linear regression and expected concentration was 0.9952 (above 0.95) (Figure 5A), which verified the credibility of the experiment. The results indicated that neither the empty plasmid nor that containing the c.-4C>T variant presented a significant difference in enzyme activity compared to that of the WT. However, the c.649_651del and c.1048C>T variants resulted in an extreme decrease in enzyme activity. Although the c.-4C>T and c.649_651del variants caused a similar decrease in protein expression, c.649_651del showed a more severe reduction in enzyme activity than did c.-4C>T (Figure 5B). It was also shown that the enzyme activity of both the c.649_651del variant and c.1048C>T was about 70% of that of the WT. Combined with the results of the above functional experiments, the pathogenicity classification of the c.649_651del and c.1048C>T variants could be upgraded to “likely pathogenic” and “pathogenic”, respectively, according to ACMG guidelines. Nevertheless, c.-4C>T is considered a hypomorphic variant because of the reduction in *ASS1* expression without influencing enzyme activity.

## 4 Discussion

To date, only a few sporadic cases of CTLN1 have been reported in the Chinese population. In the present study, the severity of the phenotype varied greatly among the three patients. Patient 3 died 3 days after birth because of an ultrahigh ammonia level. With our follow-up data, although Patients 1 and 2 manifested elevated citrulline levels with symptomatic treatment, they displayed normal growth without apparent clinical symptoms; this has rarely been mentioned in previous reports and may guide the prognosis of patients with CTLN1.

Five variants from three families were identified, of which the variant c.649_651del (p.P217del) leads to an amino acid loss within the synthetase domain. Previous studies have shown that mutations in the structural domain of the synthase can affect enzyme activity and reduce the affinity to substrates (aspartate and citrulline) (DIMMOCK et al., 2009). We found that the enzymatic activity of the c.649_651del variant was significantly impaired. In the other novel variant, c.1048C>T, the glutamate-350 codon (CAG) is converted to a nonsense codon (TAG).ENGEL et al. (2009) reported a German CTLN1 patient with a nearby nonsense mutation c.1030C>T (p.R344*).MILLER et al. (2014) also reported the case of a classic CTLN1 patient with a c.1069C>T (p.G357*) nonsense mutation. According to the three-dimensional structures of ASS1, the long C-terminal arm region (Gln^360^ to C-terminal) reaches the small domains (*α*-helix H3 a loop *α*-helix H4) with its C-terminal *α*-helix interacting with the ATP-binding site (DURON et al., 2019). The absence of a C-terminal arm affects the binding of ATP and prevents the formation of arginosuccinate from citrulline and aspartic acid. In our study, the c.1048C>T variant showed a significant decrease in protein levels and enzyme activity but a remarkable increase in transcription levels. We hypothesized that a negative feedback mechanism may trigger *ASS1* transcriptional compensation due to a drastic decrease in protein and enzyme activity.

To date, only two variants in the 5′UTR region have been reported in the HGMD (c.-5-10C>G and c.-4C>T), both of which have not been confirmed by functional studies and are classified as a variant of uncertain significance. Our functional studies showed that the −4C>T variant reduced both transcriptional and protein levels but did not affect enzyme activity. This may be a hypomorphic variant with reduced RNA and protein expression, and only individuals carrying another strong pathogenic variant are affected. Further exploration and long-term follow-ups are necessary to determine whether the c.-4C>T variant has other effects.

Due to NBS, an increasing number of neonates with elevated citrulline levels of uncertain clinical significance are being identified. Analysis of the *ASS1* gene can be used to confirm CTLN1 and, increasingly, to infer phenotypic severity (CHOY et al., 2016). The mutation c.1168G>A (p.Gly390Arg) in exon 15 accounts for up to 62% of alleles in European patients diagnosed with the disorder, and it is, therefore, the most common mutation in the classic form (DIEZ-FERNANDEZ et al., 2017). c.421-2A>G is the most common mutation in East-Asian countries, including Korea and Japan (LEE et al., 2013). In the present and previous Chinese CTLN1 cases, mutations showed a scattered distribution without high frequency or hotspot variant sites (Figure 2C). The most common mutation in East Asia, c.421-2A>G, has never been detected in the Chinese population.

Lin et al. (2019) concluded that p.Arg127Gln, p.Arg265Cys, p.Gly324Ser, and p.Arg363Trp are associated with a severe phenotype, whereas p.Ser18Leu, p.Val141Gly, p.Pro144Arg, and p.Cys337Arg may allow for some residual ASS1 function. In the present study, the c.1048C>T variant was identified in two unrelated patients. Patient 3 died 3 days after birth, and the citrulline levels in patient 1 were high even after treatment. Thus, we hypothesized that the c.1048C>T variant induces a severe phenotype. Another trans c.847G>A variant in patient 3 has been reported in several other patients and also caused a severe phenotype; therefore, the combination of c.1048C>T and 847G>A variants probably resulted in poor prognosis or rapid progression in Patient 3. Meanwhile, we find that the peak blood ammonia levels and citrulline concentrations differed significantly among the three patients in this study. It has been reported in the literature that residual ASS1 activity correlates with these biochemical indicators (ZIELONKA et al., 2019). Using reported models for prediction, residual ASS1 activity was estimated to be 10%–30% of control in patient 1, 70%–80% in patient 2, and less than 8.1% in patient 3. However, in our ELISA experiments, the enzyme activities of both the c.649_651del mutation and c.1048C>T were around 70% of the wild type, Although the trend of decreasing enzyme activity was consistent, it was much higher than expected. We speculate that there may be two reasons contributing to these differences. 1) We measured the enzyme activity of each mutation and have not yet measured the actual enzyme activity of the compound heterozygous combination, which differs from the mammalian biallelic expression system in the published article. We speculate that the combination of mutations may produces a stacking effect and aggravates the impairment of the enzyme activity. A previous study reported that the spectrophotometric analysis of the *ASS1* enzyme can properly assess the actual enzyme activity of the compound heterozygous combination, which can be used in our further research (ZIELONKA et al., 2019). 2) In our study, we used ELISA to determine the enzyme activity. This method relies on the specificity of antibodies. When the specificity of antibodies is insufficient, they will bind or react with non-specific proteins, which would reduce the sensitivity of the assay and eventually lead to higher enzyme activity.

Considering the clinical heterogeneity of CTLN1, more functional research on the variants of *ASS1* and clinical data are needed to elucidate the genotype-phenotype correlation in patients with CTLN1. We will also endeavor to collect more cases of CTLN1 patients to help provide clear incidence and prevalence statistics of CTLN1 in the Chinese population.

## 5 Conclusion

In this study, we described three Chinese CTLN1 families and identified two novel variants, c.649_651del (p.P217del) and c.1048C>T (p.Q350*), in *ASS1* gene, which were classified as pathogenic or likely pathogenic after functional validation. Another non-coding variant, -4C>T, is considered to be a hypomorphic variant. Our findings enrich the spectrums of mutations in the *ASS1* gene and, as well as contribute to the definitive diagnosis and genetic counselling of CTLN1.

## Data Availability

The data presented in the study are deposited in the open database Genome Sequence Archive in National Genomics Data Center (https://ngdc.cncb.ac.cn/gsa-human/) and the accession number is HRA003542.
