# Supplementary material for: Functional identification of two novel variants and a hypomorphic variant in ASS1 from patients with Citrullinemia type I
Source: Front Genet. 2023 Jul 7;14:1172947. doi: 10.3389/fgene.2023.1172947 (PMC10360398; doi:10.3389/fgene.2023.1172947)
Supplement: Supplementary file 1 [file Table1.DOCX]

Supplementary Material

Functional identification of two novel variants and a hypomorphic variant in *ASS1* from patients with Citrullinemia type I

**Jing Liu^1,2,^** †**, Zhongjie Wang^3,^** †**, Huiming Yan^1^, Yanling Teng^3^, Qingxin Shi^3^, Jing Chen^1^, Wanglan Tang^1^, Wenxian Yu^1^, Ying Peng^1,2^, Hui Xi^1,2^, Na Ma^1^, Desheng Liang^3,4^, Zhuo Li^3^*, and Lingqian Wu^3,4^***

^1^Department of Medical Genetics, Maternal and Child Health Hospital of Hunan Province, Changsha, Hunan, 410008, China.

^2^National Health Commission Key Laboratory of Birth Defects Research, Prevention and Treatment, Changsha, Hunan, 410008, China.

^3^Country Center for Medical Genetics, Hunan Key Laboratory of Medical Genetics & Hunan Key Laboratory of Animal Models for Human Diseases, School of Life Sciences, Central South University, Changsha, China. 410078

^4^Country Laboratory of Molecular Genetics, Hunan Jiahui Genetics Hospital, Changsha, Hunan, China. 410078

*** Correspondence:**

Center for Medical Genetics, Hunan Key Laboratory of Medical Genetics & Hunan Key Laboratory of Animal Models for Human Diseases, School of Life Sciences, Central South University, Changsha, Hunan 410078, China. E-mail: [lizhuo@sklmg.edu.cn](http://lizhuo@sklmg.edu.cn)

Lingqian Wu, Ph.D., M.D.

Center for Medical Genetics, Hunan Key Laboratory of Medical Genetics & Hunan Key Laboratory of Animal Models for Human Diseases, School of Life Sciences, Central South University, Changsha, Hunan 410078, China. E-mail: [wulingqian@sklmg.edu.cn](http://wulingqian@sklmg.edu.cn)

# Supplementary Figures and Tables

##
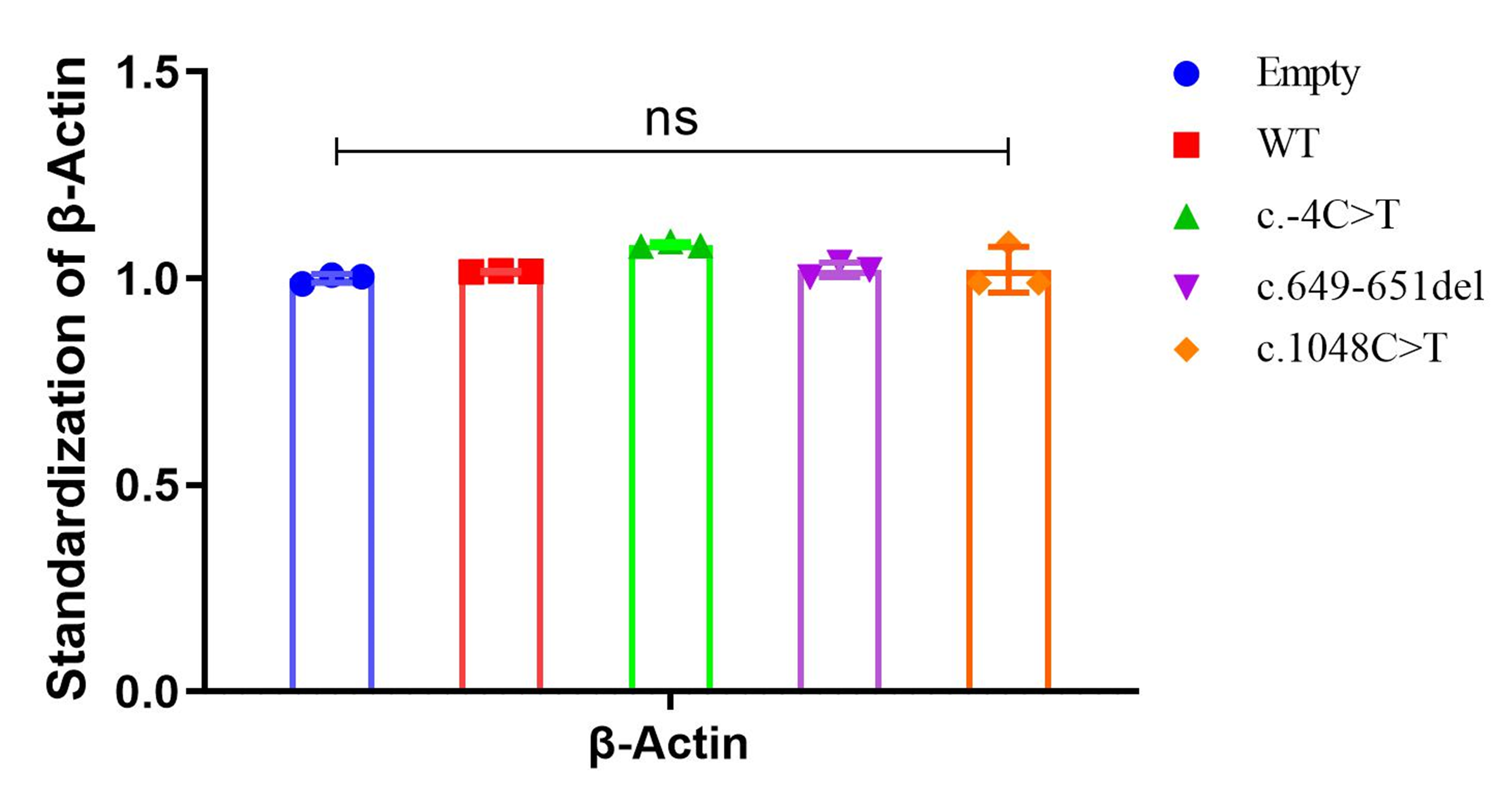
Supplementary Figure 1

**
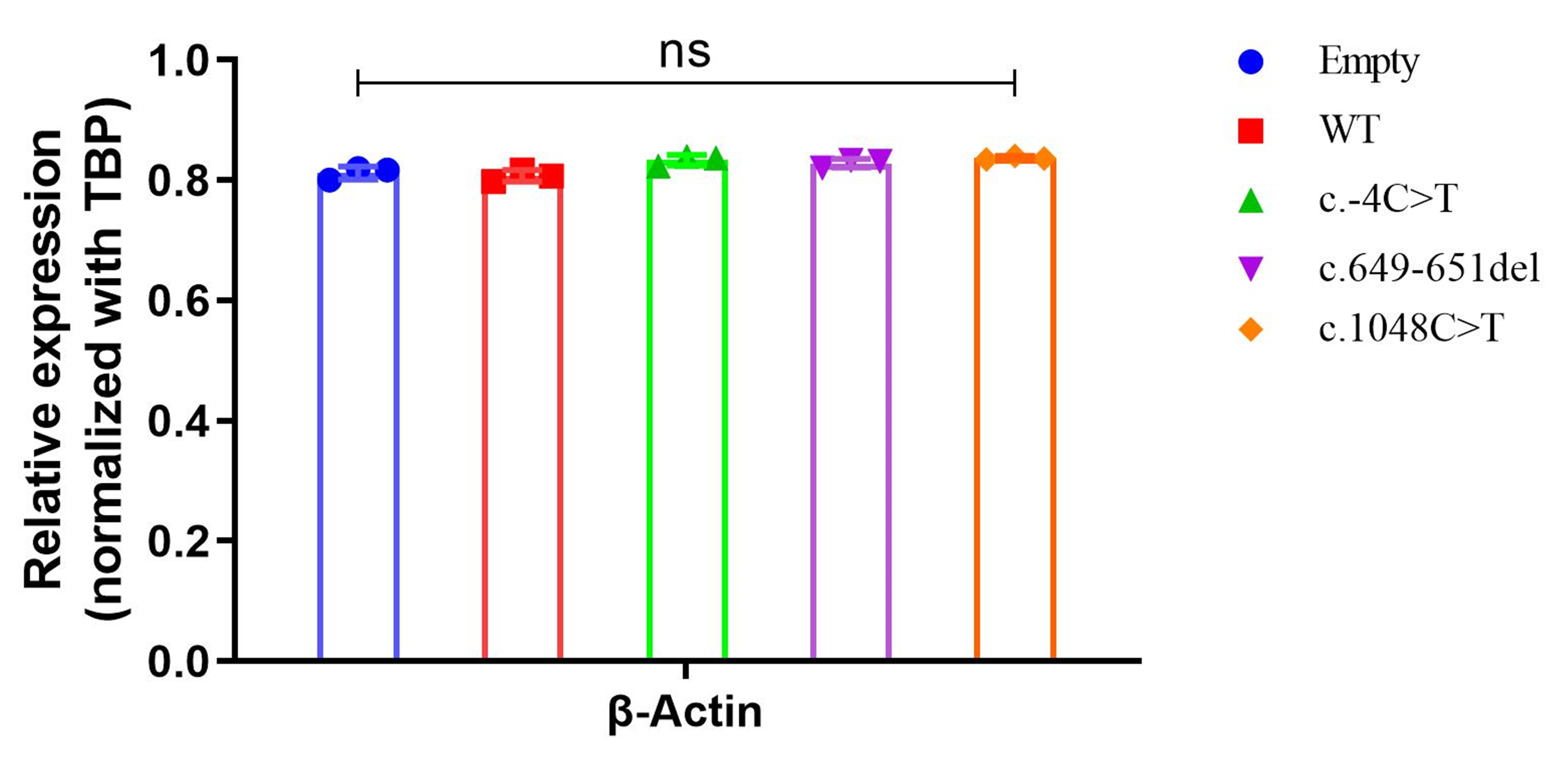
** **A
 B**

**
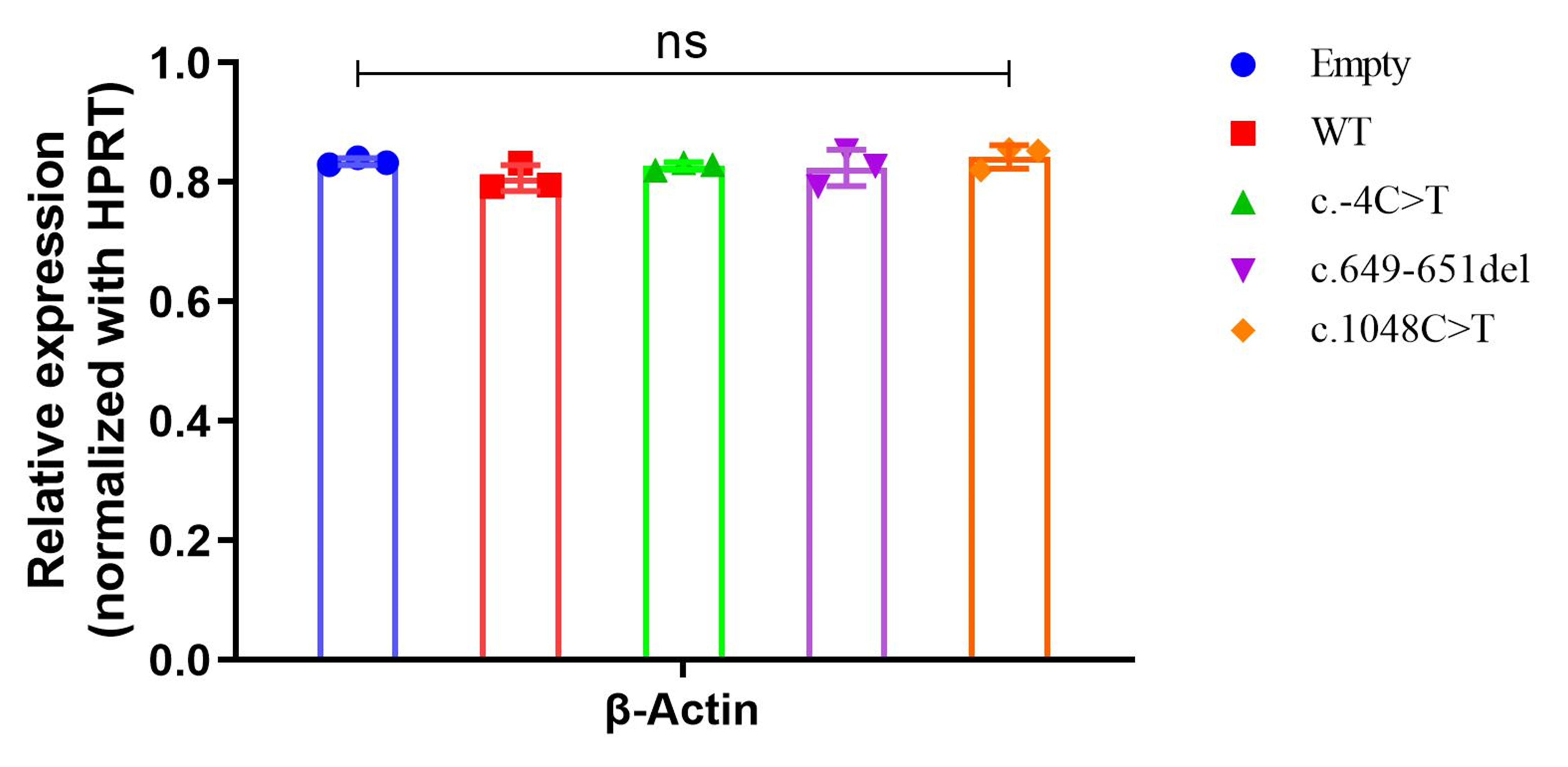
**

**C**

**Supplementary Figure 1.** Standardization of *β-Actin*. A-C: Three housekeep genes *β-Actin*, *TBP* and *HPRT* were normalized by *β-Actin* in different groups, respectively. There was no difference between the different groups when standardizing the internal reference.

## Supplementary Figure 2


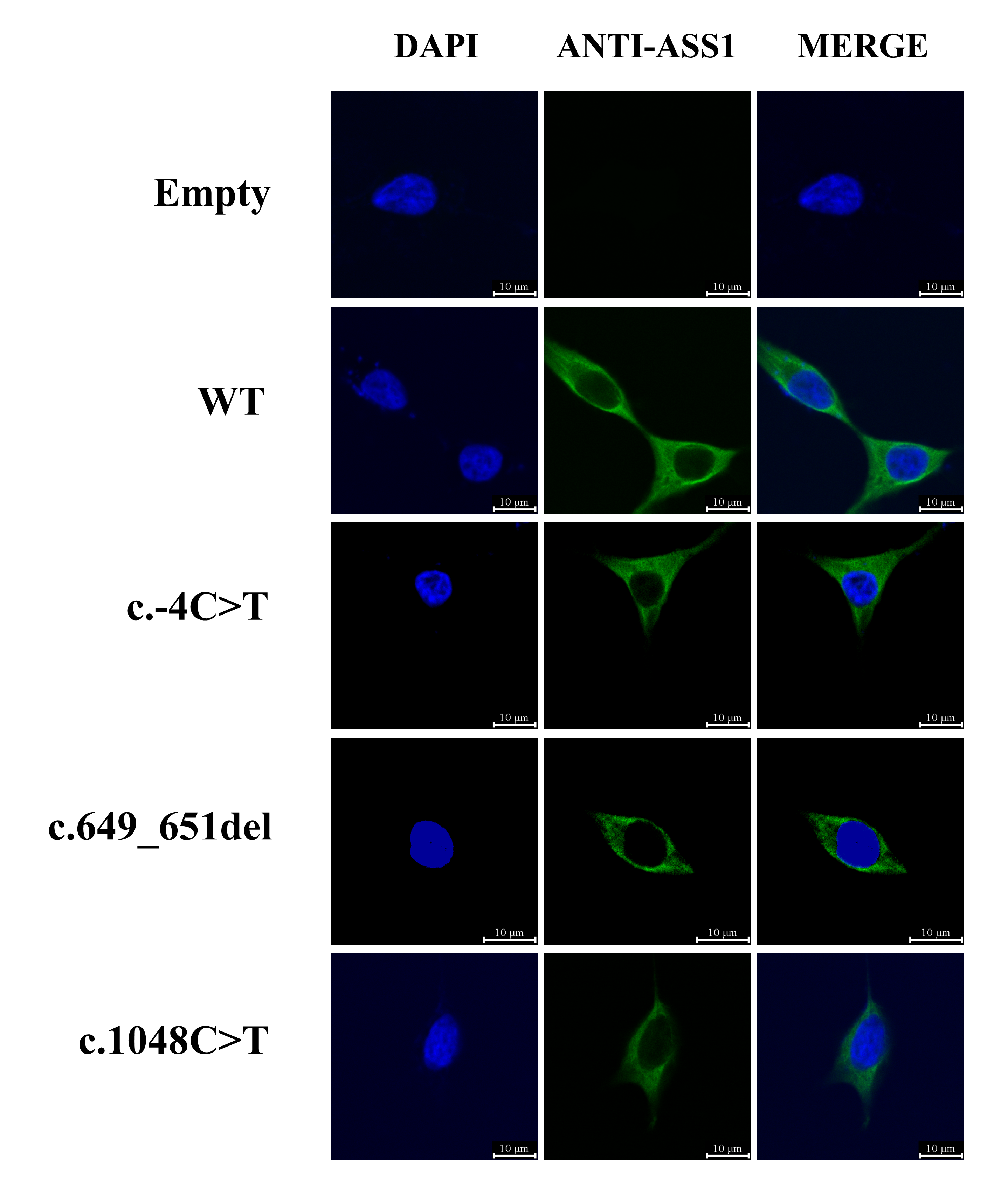


**Supplementary Figure 2**. Representative confocal microscopy images of WT or mutant ASS1 plasmids transfected into 293T cells. Both wild-type and mutant ASS1 proteins are localized in the cytoplasm without significant differences. Scale bars represent 10 µm.

## Supplementary Table 1

**Site-directed mutagenesis primers**

| Name | Sequence (5′→3′) |
| --- | --- |
| ASS1-4CT-F | GCCAGAtGCTATGTCCAGCAAAGGCTCCGTGG |
| ASS1-4CT-R | TGGACATAGCaTCTGGCGGGAGTCGAGGAACA |
| ASS1-649_651del-F | CAACACCCCTGACATTCTCGAGATCGAGTTCAA |
| ASS1-649_651del-R | AGAATGTCAGGGGTGTTGGCTTTGGCTGGGTCCTG |
| ASS1-1048CT-F | AAGGGAAAGTGAGGTGTCCGTCCTCAAGGGCCA |
| ASS1-1048CT-R | GACACCTCACTTTCCCTTCCACTCGCTCCTGGG |

## Supplementary Table 2

**The processing of qPCR data**

| Sample | Independent tests | Ct | | | | 2^-△△Ct | | |
| --- | --- | --- | --- | --- | --- | --- | --- | --- |
|  |  | *β-Actin* | *ASS1* | *TBP* | *HPRT* | *ASS1-*  *β-Actin* | *TBP-*  *β-Actin* | *HPRT-*  *β-Actin* |
| WT | 1 | 20.23 | 17.98 | 22.44 | 22.34 | 1.00 | 1.00 | 1.00 |
|  | 2 | 20.57 | 18.35 | 22.43 | 22.43 | 0.98 | 1.27 | 1.19 |
|  | 3 | 20.66 | 18.48 | 22.57 | 22.38 | 0.95 | 1.23 | 1.31 |
| Empty | 1 | 20.82 | 26.60 | 22.63 | 22.54 | 0.00 | 1.32 | 1.31 |
|  | 2 | 20.87 | 26.80 | 22.73 | 22.53 | 0.00 | 1.27 | 1.37 |
|  | 3 | 20.80 | 26.62 | 22.66 | 22.58 | 0.00 | 1.27 | 1.26 |
| c.-4C>T | 1 | 20.03 | 17.95 | 22.04 | 22.02 | 0.89 | 1.15 | 1.09 |
|  | 2 | 20.06 | 18.02 | 22.18 | 22.07 | 0.86 | 1.06 | 1.07 |
|  | 3 | 20.26 | 18.38 | 22.13 | 22.01 | 0.77 | 1.27 | 1.21 |
| c.649-651del | 1 | 21.28 | 19.18 | 23.12 | 22.86 | 0.90 | 1.29 | 1.44 |
|  | 2 | 20.55 | 18.47 | 22.78 | 22.43 | 0.89 | 0.99 | 1.17 |
|  | 3 | 20.91 | 19.04 | 22.86 | 22.56 | 0.77 | 1.20 | 1.38 |
| c.1048C>T | 1 | 20.18 | 16.57 | 22.22 | 22.22 | 2.57 | 1.13 | 1.05 |
|  | 2 | 20.26 | 16.60 | 22.16 | 22.25 | 2.66 | 1.24 | 1.09 |
|  | 3 | 20.26 | 16.88 | 22.22 | 22.21 | 2.19 | 1.19 | 1.12 |

# Supplementary Protocol 1

**The protocol used for DNA amplification**

All DNA amplification reaction systems are shown in the table below:

| Reagents | Volume(μL) |
| --- | --- |
| Premix Ex Taq Hotstar | 10 |
| DNA | 1 |
| Forward primers | 1 |
| Reverse primers | 1 |
| ddH_2_O | 7 |
| Total | 20 |

All reactions are amplified according to the following conditions:


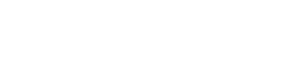

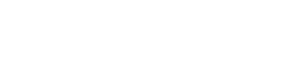

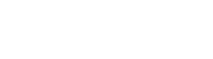

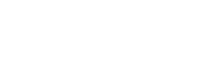

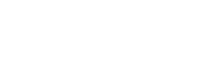

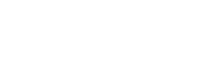

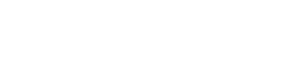


95℃ 5min

95℃ 30s

30 cycles

TM 30s

72℃ 1kb/60s

72℃ 5min

4℃ ∞
